# Experimental Study and Verification of New Monolithic Rotary Cutting Tool for an Active Driven Rotation Machining

**DOI:** 10.3390/ma15051630

**Published:** 2022-02-22

**Authors:** Andrej Czán, Richard Joch, Michal Šajgalík, Jozef Holubják, Andrej Horák, Pavol Timko, Jan Valíček, Milena Kušnerová, Marta Harničárová

**Affiliations:** 1 Department of Machining and Manufacturing Technology, Faculty of Mechanical Engineering, University of Žilina, Univerzitná 1, 010 26 Žilina, Slovakia; andrej.czan@fstroj.uniza.sk (A.C.); richard.joch@fstroj.uniza.sk (R.J.); michal.sajgalik@fstroj.uniza.sk (M.Š.); jozef.holubjak@fstroj.uniza.sk (J.H.); andrej.horak@fstroj.uniza.sk (A.H.); pavol.timko@fstroj.uniza.sk (P.T.); jan.valicek@fstroj.uniza.sk (J.V.); 2 Department of Mechanical Engineering, Faculty of Technology, Institute of Technology and Business in České Budějovice, Okružní 10, 370 01 České Budějovice, Czech Republic; kusnerova.milena@mail.vstecb.cz

**Keywords:** turning, rotary tool, cutting force, actively driven tool, surface texture

## Abstract

Forced rotation turning appears to be an effective machining method due to higher tool life, time efficiency and acceptable quality. Several studies have been carried out to investigate the basic characteristics of forced rotation machining. So far, tools are used whose design included several components. However, such tools may generate vibrations, which are undesirable in the process. In engineering practice, most vibration problems are solved by reducing the cutting parameters (cutting speed and feed rate), which reduces machining productivity. For this reason, a new type of monolithic rotary tool has been designed that eliminates the design complexity and high assembly accuracy requirements of current rotary tools. Based on the performed experimental research, it is possible to define the influence of cutting parameters on the cutting force. Next, the equation of the cutting force and the resulting roughness of the machined surface was determined. In the introduction, the results of the analysis of machining parameters with a rotary tool were added. The presented solution fundamentally validates the new monolithic tool for forced rotation technology and defines its application for different machining materials.

## 1. Introduction

**Figure 1:**
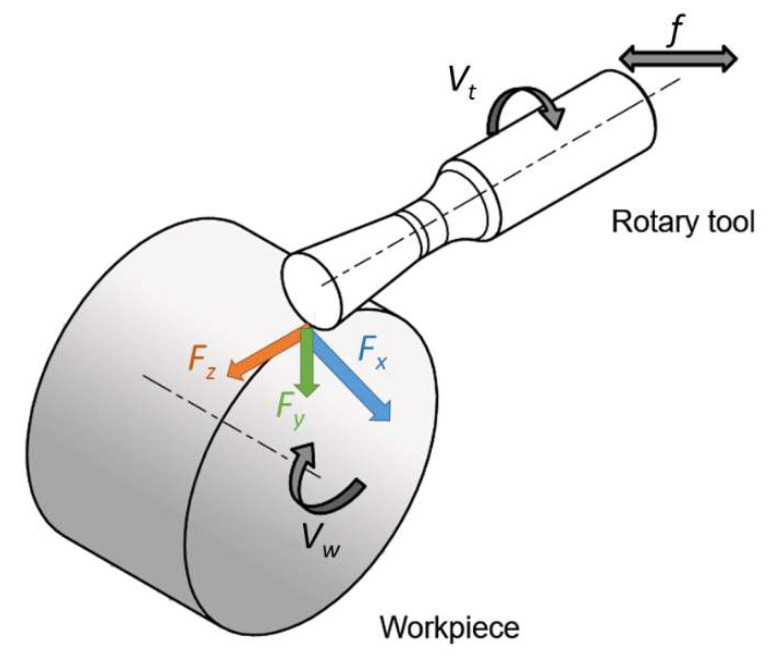
Kinematic diagram of ADRT.

There is still a lot of production activity in the field of machining, and so there is a need for continuous improvement in this area. Rotary tool machining is considered one of the usable methods for future machining. It can be applied in the process of milling, planing and turning of materials with enhanced mechanical properties [1,2]. Based on the mode of rotation occurrence in a rotary tool in the machining process, we know two basic modes of rotation, namely, a tool with constrained rotation (SPRT) and a tool with forced rotation (ADRT) [1,3,4]. Tools with constrained rotation have a rotational motion derived from the friction forces generated between the workpiece and the tool contact surfaces [5]. This type of tool does not require a separate drive for its rotation [6]. On the other hand, tools with forced rotation have rotary motion provided from an external drive. The latter does not have to be part of the machine tool, especially in the case of conventional machine tools [7]. If the technology is used for CNC (Computer Numeric Control) machines, the CNC machine can provide the tool drive. However, the machine in question must have the possibility to machine-driven tools, for example, directly or with the help of a driven tool holder. The machining productivity highly depends on the tooling material used and its cutting and working properties, especially when machining materials with increased mechanical properties [8]. Machining of refractory steels and alloys is associated with high cutting forces and considerable thermal loading of the cutting edge of the tool. In order to ensure machining efficiency, tools must meet high cutting wedge strength and resistance to thermal stresses. It should be noted that turning tools made from high-speed steel and sintered carbides may not always meet the above two requirements. An option is applying ceramic cutting inserts, which have a good service life but a high price [9]. Different designs of rotary circular tools can be a solution to these requirements due to the use of different kinematic cutting schemes (Figure 1). The setting of parameters such as workpiece rotation speed (*v_w_*), tool rotation speed (*v_t_*), feed rate (*f*) or the direction of cutting force components (*Fx*, *Fy*, *Fz*) is different in such kinematic schemes compared to standard turning. The tool is cooled to a greater extent compared to conventional methods due to the effect of its rotation [10]. The rotation of the cutting part of the tool arranges a better heat dissipation around its circumference due to the contact of only one point of the cutting edge at each time [11]. Nguyen et al. analysed the machining process with rotary tools and machined quality of the rotary turning of the hardened steel materials under a variety of machining parameters. The highest values of depth of cut (0.6 mm), cutting speed (200 m·min^−1^) and feed rate (0.6 mm) can be used to increase energy efficiency. The lowest values for depth of cut (0.2 mm) and feed rate (0.2 mm) are recommended to reduce roughness. The highest level of cutting speed can be used to achieve a smoother surface [12]. The main difference from auto-rotating tools, whose rotational motion is dependent on the machining parameters, is that in tools with forced rotation, the frequency of rotation can be controlled, and its character can be changed with the help of an external actuator. Thus, it is realistically possible to test several options and arrange possibly better productivity than auto-rotating tools.

Some practical tests of forced-motion tools have shown that the ability to handle mechanical and thermal loads on the tool were surprisingly effective compared to conventional machining methods. Available reported records speak of up to 400 to 500% higher productivity and up to 1500 to 2000% higher tool life. According to the available test information, it has been found that radial forces decrease with increasing tool rotational speed, while axial forces increase with increasing tool speed [4]. When machining with a tool with forced rotation, two basic options of rotary tool motion can be used in the cutting process. The rotation can be carried out in clockwise and counterclockwise directions [5]. In the counterclockwise orientation, observations have shown that the tool actually moves from the location where the chip is formed to a greater extent to the location with fewer chips. More surface imperfections were observed by machining in counterclockwise rotation and by increasing the rotational speed of the tool used. Another parameter that affects the machining process by forced rotation is the angle of inclination of the cutting edge to the workpiece axis (λs). In forced rotation, this angle does not fundamentally affect the rotational speed of the tool, as it is provided by an external drive unit. For a correct setting, it is necessary to investigate λs, since at certain values, there can be a critical increase in cutting force [13,14]. High temperatures are generated at the cutting point when machining materials and overall high cutting forces are generated [15]. For this reason, conventional tools must be designed to have high cutting wedge strength and also cutting wedge resistance to high temperatures. However, the problem with conventional tools still remains the fact that in the machining process, the same part of the tool is always worn on the tool, which will mainly affect the machining quality and durability of the tool [4,10,16]. For a better understanding of the forced rotation machining process, it is necessary to know the force loads at different rotation settings. It can be assumed that the direction of rotation alone will have different results for different machined materials. Cutting tool manufacturers have very limited options for eliminating unwanted vibrations; these include cutting tool geometry, tool body material and, where appropriate, cutting tools with integrated vibration damping mechanisms. In the presented paper, the elimination of unwanted vibrations is addressed by a non-standard cutting tool geometry.

## 2. Cutting Tool

### 2.1. Desing of the Rotary Tool

**Figure 2:**
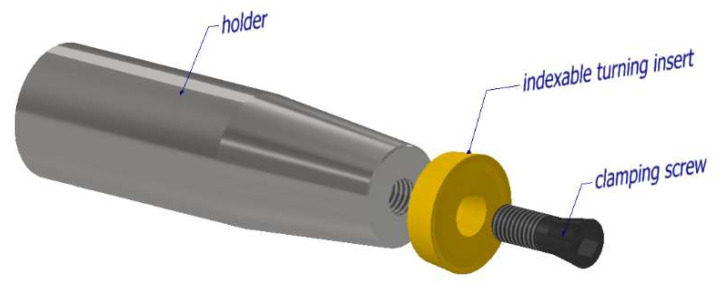
Design of rotary tool.

**Figure 3:**
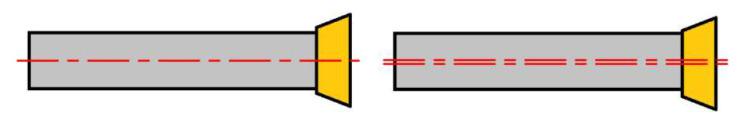
Inaccuracy of the cutting insert fit against the clamping part.

In the scientific studies carried out, tools were used whose cutting part consisted of a standard available replaceable cutting insert (Figure 2) [4,5]. It should be noted that these cutting inserts are not designed for machining with active rotation, and their clamping system corresponds to this. When using a replaceable cutting insert of circular shape, high precision of the insert fitting to the clamping part is necessary. If the conditions of high precision are not met, misalignment can occur (Figure 3), which can result in unwanted vibrations during the machining process.

**Figure 4:**
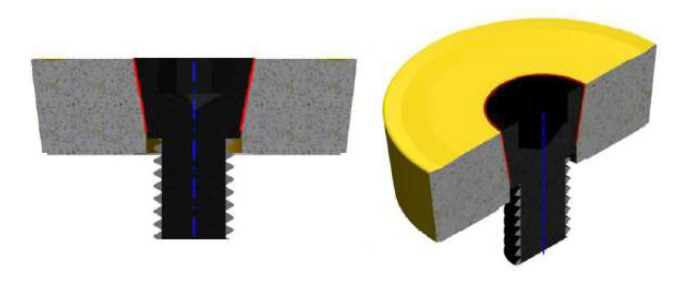
The tapered screw clamping mechanism of the cutting insert.

Strict requirements for accuracy of fit can be met by the use of clamping screws whose head has a negative shape of the cutting insert hole. With tapered shapes, partial concentricity of fit can be achieved (Figure 4). Such a solution is costly because every single type of cutting insert must have a specially manufactured clamping screw.

### 2.2. Monolithic Rotary Tool

**Figure 5:**
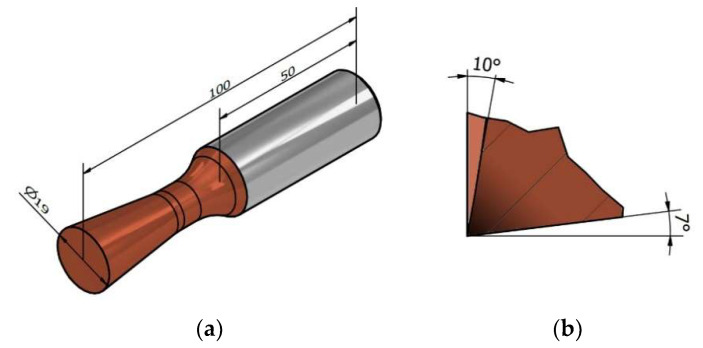
New monolithic tool: (**a**) basic dimensions of the tool; (**b**) geometry of the cutting wedge.

When turning with circular interchangeable cutting inserts, a cutting force is generated that requires stable holder clamping, clearance definition, and sufficient rigidity. Standard clamping systems require a clamping taper with a rounded hole, and the clamping screw must be in line with the axis of the cutting insert and tool holder. With this design, vibrations in the machining process may occur due to incorrect positioning of the cutting insert due to the concentricity of the insert and tool holder. For this reason, a new monolithic cutting tool (Figure 5) was designed to eliminate the shortcomings of the cutting insert mounting. The cutting portion of the tool was dimensioned to 19 mm, and the tool geometry was defined based on previous experimental findings. The face angle was set at 10° and the relief angle was 7°. The design of the presented monolithic tool was filed in 2020 at the Industrial Property Office of the Slovak Republic as a patent solution.

**Figure 6:**
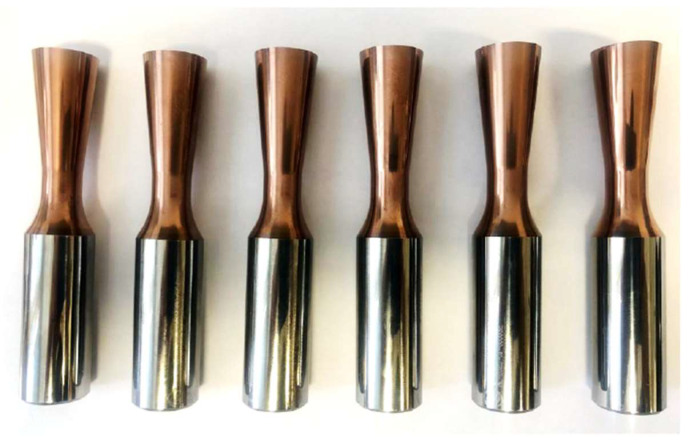
Monolithic rotary tools for forced rotation machining.

The monolithic rotary turning tool is made of sintered carbide with a 10% cobalt content for high tool life and the ability to machine relatively hard and difficult to machine materials. The tool must maintain cutting edge sharpness even at high speeds and should also be able to withstand multiple experimental settings of machining parameters. The tapered shank of the monolithic tool is designed for better knife alignment capability with respect to the workpiece and to prevent unwanted excessive friction of the tool rotary surface against the workpiece surface during machining. In order to experimentally validate the monolithic rotary tool in the machining process, six tools with identical geometry and material composition were fabricated (Figure 6).

## 3. Materials

Identification of the applicability of the monolithic tool was carried out on several types of materials with different mechanical properties. The aim was to determine materials that would represent a group of tough and difficult to machine materials. 

### 3.1. Material to Be Machined—Tool Steel

90MnCrV8 is a cold-forming tool steel with high form stability in heat treatment, very high cracking resistance, high machinability, medium toughness and wear resistance. Its chemical composition is given in Table 1 [17].

After hardening, a hardness of 63 to 65 HRC (Hardness Rockwell C) can be achieved. In addition, tempering to a different temperature provides a wide range of possible workpiece hardness: from 38 HRC with tempering at 600 °C to 63 HRC, tempering at 100 °C [17]. For the experiment, the workpiece was heat treated to the desired hardness of 58 HRC. This material is predominantly used in the manufacturing of machine components such as press moulds, cold shear tools, screw taps, reamers, springs, plastic moulds, measuring and inspection tools and gauges [18]. 

### 3.2. Material to Be Machined—Aluminium Alloy

AlZn5.5MgCu is one of the strongest aluminium alloys with a tensile strength of up to 600 Mpa (often referred to as aerospace duralumin), has the highest strength and hardness of all alloys and is also characterised by excellent polishability [19]. It is mainly used where a combination of high strength and low weight is important and for highly stressed structural parts. Its chemical composition is given in Table 2.

This material is applied in a cured state. It has reduced corrosion resistance, is well machinable, short chips are produced during machining. It achieves a hardness of approx. 135 to 161 HBW (Brinell hardness). Welding is very difficult, or even impossible, also not suitable for anodising. In technical practice, it is used wherever a combination of low weight and high strength is desired, i.e., mainly in the aerospace and automotive industries, for injection moulds and moulding of plastics, gears, shafts and control valve parts. 

### 3.3. Material to Be Machined—Refractory Steel Alloy

42CrMo4 (Table 3) belongs to the group of heat-resistant low-alloy steel. It is widely used in the engineering industry. It has high fatigue strength, abrasion resistance, toughness and torsional strength. It can be heat treated in many ways to provide a combination of desired properties. It is suitable for quench hardening and tempering. After quench hardening, it forms a rigid martensitic structure due to the elements Cr and Mo, which also increase the mechanical properties of the steel such as strength, ductility and toughness [20].

This material is slightly corrosion resistant due to its low chromium content. In the annealed state, it is well machinable [21]. After heat treatment, machining is most often limited to finish grinding. It is conditionally weldable due to its high susceptibility to cracks. It is ductile in the annealed state [22], but more force is required in forming because the steel is harder than conventional carbon steel. In engineering practice, it is widely used for its good mechanical properties, especially for the manufacturing of statically and dynamically stressed components. In the automotive and aerospace industries, it is used for the manufacturing of gears and crankshafts [20], also in the oil and gas industry it is used for the manufacturing of large machinery and machine parts, fasteners, shafts, steel couplings, and for the ejectors.

## 4. Experimental Part

The core of the presented paper is the investigation of the cutting force. Generally speaking, cutting force, in particular, gives reliable information about the total amount of generated process heat, and from an energy point of view, it is one of the most sensitive indicators of machining performance. The knowledge of the magnitude of the cutting force and its behaviour during the cutting process enables follow-up of final economic-optimisation analyses of production processes, technologies and the choice of cutting tools, machine tools and cutting conditions. The presented paper also deals with the research of the generated surface because the dynamic loading of the machine-tool-workpiece system by the cutting forces also has a major influence on the stability of the cutting process, i.e., on the accuracy of the workpiece, and finally, on the quality and integrity of the machined surface.

### 4.1. Experimental Setup

**Figure 7:**
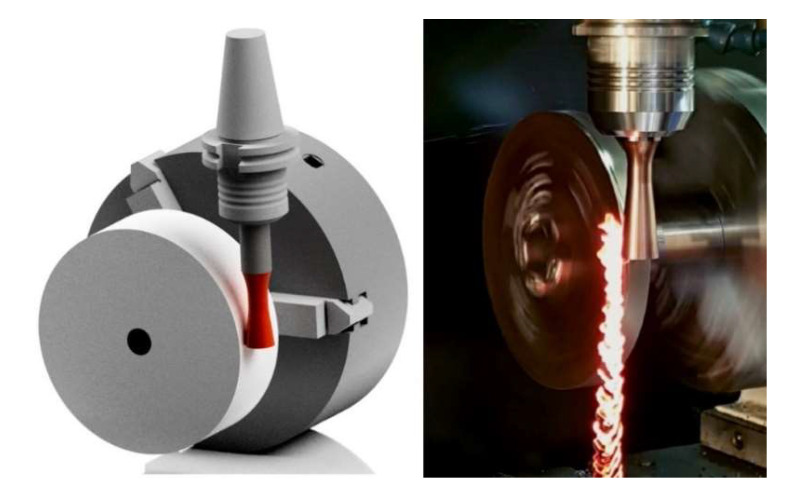
Visualisation of the tool setup and photo from the experimental machining process of a monolithic rotary tool.

The machining process monitoring with a monolithic rotary tool for turning with forced rotation (Figure 7) was carried out on a Hurco VMX 30 T machine (Hurco North America One Technology Way P.O. Box 68180 Indianapolis, IN 46268), in which the tool is clamped in the spindle and can be positioned in the *x*-, *y*-, *z*-axis. The given machine does not allow tilting of the knife inclination and thus does not create the possibility for testing the angle *λs*. The monolithic knife is clamped in the controlled spindle of the machine, and the workpiece rotates by means of a KITAGAWA rotary (Kitagawa Europe Ltd Unit 1, The Headlands Downton Salisbury Wiltshire SP5 3JJ United Kingdom, Salisbury, UK) table located on the worktable of the machine tool. 

The spindle of the KITAGAVA table did not allow the use of higher workpiece speeds than 25 min^−1^, which does not allow a more comprehensive verification of the potential of the knife technology in the experimental work. The workpieces of the materials in question should initially test the suitability of introducing this technology using a monolithic knife in the area of further research.

### 4.2. Selection of Cutting Parameters

On the basis of pilot verification of the forced rotation machining process, the basic cutting parameters were determined, which were determined according to previous experimental research [7]. The workpiece rotation speed was limited by the maximum speed of the workpiece spindle, which was set up at 10 m·min^−1^. The setting of cutting parameters was different in previous studies [6,12,15,23]. For this reason, it is necessary to rely on previous knowledge and control cutting parameters based on the results. 

However, the values of the basic cutting parameters, depth of cut and feed rate were already set in the pilot study to achieve the relatively lowest possible roughness. The objective of increasing energy efficiency is linked to setting the cutting speed as high as possible and the need for tool cooling due to the higher friction. Today’s technical practice focuses mainly on the production of high-precision and high-performance monolithic tools and develops adequate tooling suitable especially for higher-performance CNC machine tools (e.g., the German company Dietrich Karnasch, Görsdorf, Germany).

The experiments were carried out at the specified cutting parameters so that every single material was machined and then evaluated. For every single material, at constant feed parameters and depth of cut, the design cutting speed was varied, as shown in Table 4.

### 4.3. Measurement of Cutting Forces

During the machining process, three perpendicular components of the cutting force were measured using a stationary dynamometer (Table 5). This is a measuring device designed for chip machining processes. It basically works on the principle of the piezoelectric effect, where the mechanical load on the piezoelectric sensor generates an electrical charge. The dynamometer is equipped with four sensors. Each sensor is piezoelectric and has three components, compressive and tensile forces in the *Fz* direction positive and negative shear forces in the *Fx* and *Fy* directions, while the orthogonal force components are recorded simultaneously. For the 3-component measurement during machining, eight signals leading to the hub amplifiers are obtained. These contain a capacitor, and by changing its range, the range of the measured force is changed. The amplified charge signal is converted to a voltage signal which is then evaluated. The magnitude of the charge or electrical voltage is proportional to the magnitude of the applied force [24].

**Figure 8:**
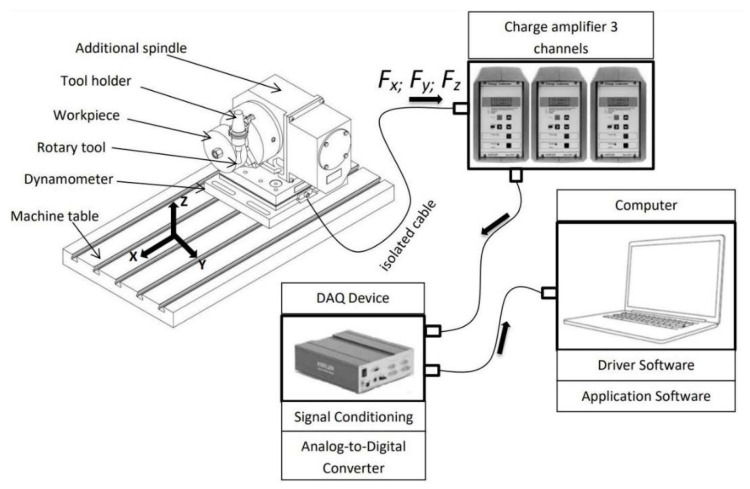
Schematic wiring of the cutting force component measurement assembly.

A Kistler 9255A stationary piezoelectric dynamometer was used to measure the cutting force components during turning with powered tools. The dynamometer was mounted on the table of the used machine tool. A rotary head with a chuck (Figure 8) was mounted on the dynamometer, in which the collet with the workpiece was clamped. The monolithic tool was clamped in the spindle of the machine tool and can be positioned in *x*, *y*, *z* axes.

### 4.4. Surface Measurement

**Figure 9:**
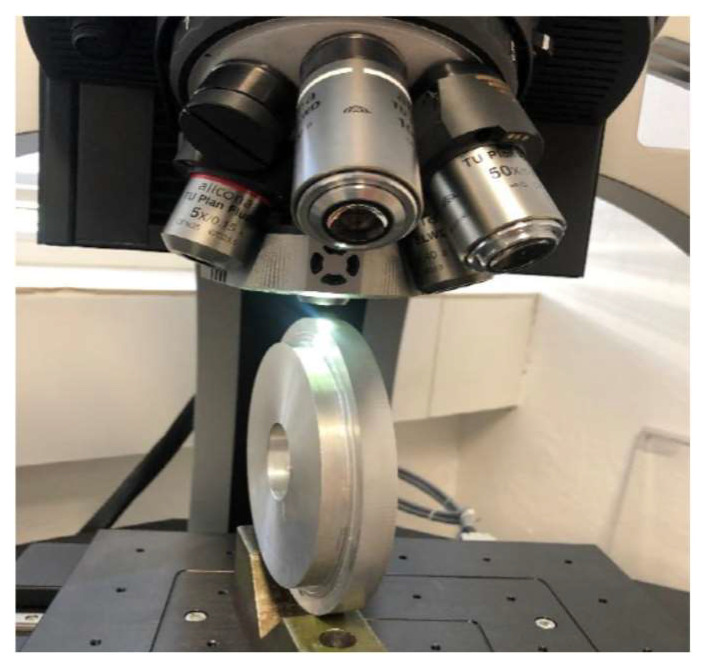
Setting up the measured sample.

The Alicona InfiniteFocus optical system was used to evaluate the surface of the machined surfaces. This measurement system allows microscopic measurement of the coordinates and roughness of the scanned surfaces. For the purpose of roughness measurement, measurement with sufficiently high accuracy and repeatability can be applied. The measured workpiece sample was first set up on the instrument workbench under the confocal microscope (Figure 9). After the alignment and proper illumination of the measured sample, a scan of the sample surface was performed. The scanned surface of the part was then flattened using the system, and the roughness parameters *Ra*, *Rz* were evaluated from the scanned data.

Under the defined conditions, the surface roughness was measured on the evaluated length of 4 mm for the machined surface. There were five basic length measurements for determining the roughness parameters with a length of 0.8 mm. Surface measurements were performed for all materials (tool steel, duralumin steel, quenched steel) and all cutting speeds used (100 m·min^−1^, 300 m·min^−1^, 500 m·min^−1^). The cutting parameters of the depth of cut *a_p_* = 0.2 mm and feed values *f* = 0.1 mm were maintained for all materials.

## 5. Results

The results of the measurement of cutting force components during machining are presented, the resulting cutting forces and machined surfaces are compared, and the influence of cutting parameters is assessed. 

### 5.1. Components of Cutting Force in Machining

**Figure 10:**
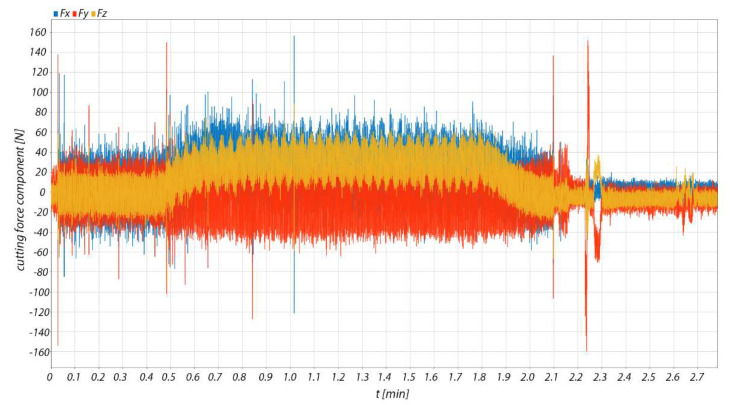
Components of the measured cutting force in machining AlZn5.5MgCu.

Figure 10 illustrates the directly measured cutting force components *Fx*, *Fy*, *Fz* for the machined material AlZn5.5MgCu. The sampling frequency of the dynamometer was 5000 Hz. The total cutting force *F* was calculated from these measured data, the values of which are then exported using Matlab R2021a (Humusoft s.r.o, Pobrezni 20, 186 00 Praha 8 Czech Republic).

As shown in Figure 10, the measurement itself captures the entire machining process from the first tool-workpiece contact to the completion of chip cutting. For this reason, only a slice of the measurement record that presents the chip machining itself was analysed.

In the case of the measurement of the cutting force components for the machined materials 90MnCrV8 and 42CrMo4, the procedure was identical. 

### 5.2. Comparison of the Resulting Cutting Forces

**Figure 11:**
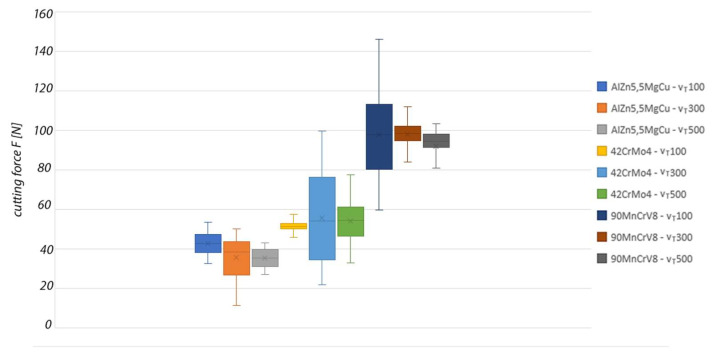
Graphical comparison of the total cutting force for each experiment.

Based on the obtained cutting force data from the machining process, a graphical comparison of the resulting cutting force in the machining process for each material and cutting speed was created. The results of the experiment are shown in Figure 11. 

The achieved cutting forces at the individual tool rotation speeds for machining AlZn5.5MgCu were in the low range up to 50 N. When the rotation speed of the monolithic tool was increased, the cutting force decreased up to 35 N at a tool rotation speed of 500 m·min^−1^.

The mean cutting force values in the individual machining experiments for 42CrMo4 were very similar. The visible difference of the individual tool rotation speed settings is in the variance of the measured force values. For a tool rotation speed of 300 m·min^−1^, it ranges from 35 N to 77 N. This variance of values is the highest of the experimental measurements performed.

When machining the hardest material, which was 90MnCrV8, the values of the total cutting force were in the range of 90 N ± 5% at a tool cutting speed of 500 m·min^−1^. Higher values of the total cutting force and also a dispersion of values were observed when machining at lower tool rotation speeds. Based on these data, we can assume that the monolithic tool can be used for machining materials with enhanced mechanical properties under properly specified cutting conditions. 

### 5.3. Comparison of the Machined Surface

**Figure 12:**
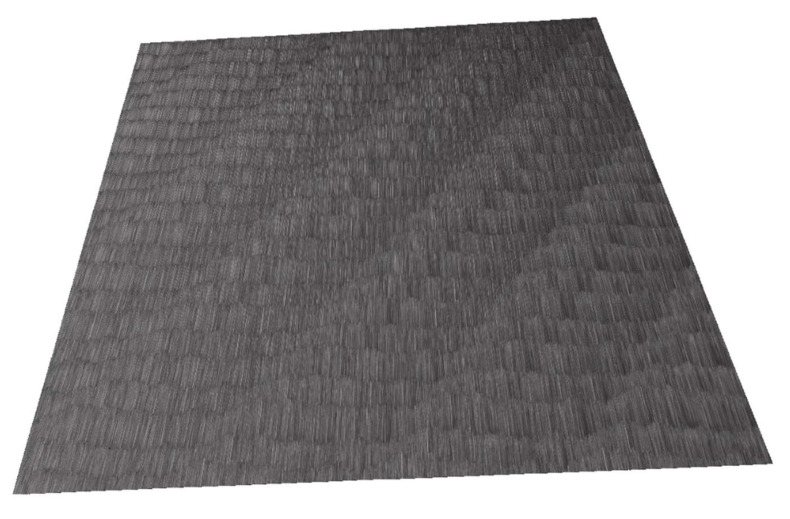
Optical surface texture measurement 42CrMo4 at cutting speed of 300 m·min^−1^.

In the image of the enlarged surface of AlZn5.5MgCu, square and hexagonal tool marks can be seen after machining under the given conditions. These marks and their character are determined by the resulting direction of the main cutting motion. The surface formed at a cutting speed of 500 m·min^−1^ was different from the other cutting speeds and had a hexagonal structure. The hexagonal features are distributed over the entire surface of the machined material and can be formed by the worn part of the tool. During rotary turning of 42CrMo4 at a cutting speed of 100 m·min^−1^, the surface showed diamond-shaped machining marks, in which elliptical traces of the circular cutting edge of the tool appeared on the colour map. The resulting cutting motion vector gives the overall orientation direction of these traces. For a steel workpiece machined with a rotary tool at a cutting speed of 300 m·min^−1^ (Figure 12), the tool marks have a striped character with fine rectangular shapes. The direction recorded by the colour map shows the vector of the resulting cutting motion. 

In the images of the surface machined at a cutting speed of 500 m·min^−1^, the layered tool marks of the rotary tool can be seen after high-speed machining. At the high cutting speed of 500 m·min^−1^, the shape of the machining marks is different for steel compared to the other two lower cutting speeds. This effect was evident in the machining of AlZn5.5MgCu. For the 90MnCrV8 material machined at a cutting speed of 100 m·min^−1^, rectangular strip formations appear on the surface, the direction of which is also determined by the vector of the resulting cutting motion. The width of the strip is defined by the knife geometry and the feed rate. Images of the machined surface at a cutting speed of 300 m·min^−1^ show finely textured rectangular layers. Their direction is determined by the resulting main cutting motion vector. The structure is finer but similar for identical parameters of 42CrMo4 steel. 

**Figure 13:**
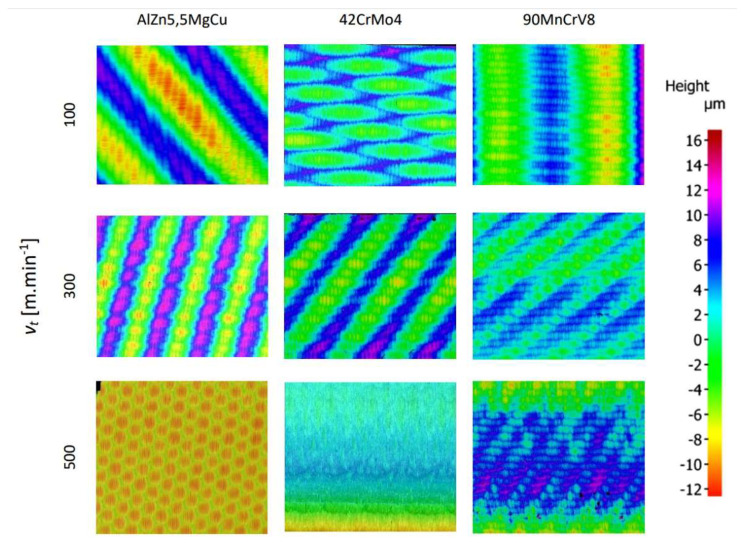
Record of machined surfaces.

**Figure 14:**
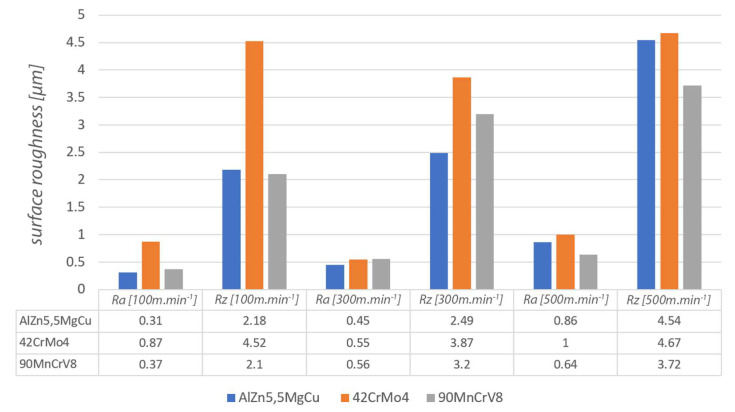
Graphical representation of surface roughness parameters.

From the recorded surfaces (Figure 13) and their roughness (Figure 14), it can be concluded that the monolithic knife and the overall technology is suitable for the implementation of high-speed machining. The machined surfaces generally have relatively low roughness values using the given parameters. By improving the overall technological system of a given process (such as increasing the frequency of rotation of the workpiece), the roughness values could be improved for the technology used.

### 5.4. Influence of Cutting Machining Parameters

In order to determine the cutting parameters, a pilot experiment was carried out to obtain basic data on the cutting force in the forced rotation machining process. The cutting parameters used were chosen within the ranges shown in Table 6 due to the system and machine stiffness. Within the design of the experiment, 56 experimental measurements were performed, where a combination of parameters from Table 6 was gradually selected. In this experiment, the effect of the rotation direction of the tool with respect to the workpiece was also investigated, where negative rotation speeds indicate a counterclockwise orientation, and positive speeds present a clockwise rotation orientation. 

The effects of the factors *v_w_*, *v_t_*, *f* and *a_p_* were analysed by the response surface method. The aim was to intensify the individual parameters and their dependence on the magnitude of the cutting force *F*. After the experiments were completed, the experimental data were subjected to statistical processing (Table 7).

The analysis of variance (Table 7) of the total cutting force *F* shows that the most significant effect is achieved by the depth of cut *a_p_*, which directly influences the size of the material removed during machining. According to the linear model, the influence of *a_p_* size on cutting force *F* reaches 24.55%. The workpiece rotation speed *v_w_* has the smallest influence on the F size of cutting force (0.20%). Moreover, the feed size *f* cannot be considered statistically significant as its influence on the change of cutting force *F* reaches the value of 0.64%. The accuracy of the mathematical model reaches 90.60%, with a prediction coefficient of 85.09%. From the above data, it is possible to construct the response surface Equation *F* (1):

*F* = 583 − 0.09 *v_w_* + 0.088 *v_t_* − 1715 *a_p_* − 1710 *f* − 0.00103 *v_w_*^2^ + 0.005289 *v_t_*^2^ + 2187 *a_p_*^2^ + 10450 *f^2^* + 0.00007 *v_w_* · *v_t_* − 0.297 *v_t_*· *a_p_* − 2.27 *v_t_*· *f* (1)

**Figure 15:**
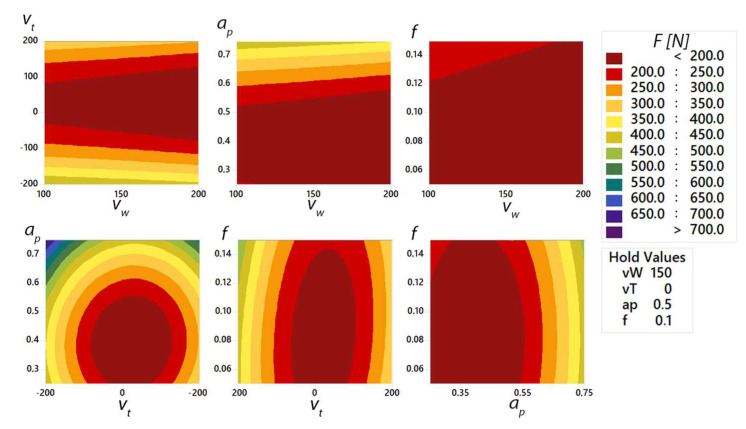
Graphical representation of the total cutting force in the machining process as a function of the individual cutting parameters.

A graphical interaction (Figure 15) of the selected cutting parameters was constructed from the experimentally obtained data and the subsequent mathematical model for machining a AlZn5.5MgCu duralumin workpiece. When the graphical representation is used, the influence of the two cutting parameters on the total cutting force *F* can be clearly observed. One of the investigated parameters was the tool rotation *v_t_*. For identical tool rotation speed and different rotation directions, the magnitude of the total cutting force is affected by 50 N. For the parameter *a_p_* = 0.75 (mm), there was an increase in the cutting force of 100 N when the direction of rotation was changed. From the above, it can be seen that the direction of rotation of the tool itself has an effect on the cutting force. 

With a properly selected tool rotation, it would be possible to apply cutting parameters (*a_p_*, *f)* to increase machining productivity compared to productivity in the opposite direction of tool rotation.

## 6. Discussion

When the given results are compared, it can be concluded that the new designed monolithic tool shows a possible application for machining a wide range of materials. The prototype design of the monolithic tool eliminates possible assembly shortcomings and inaccuracies in the manufacturing of complex tools consisting of multiple components. The main advantage is the absence of the clamping mechanism of the interchangeable cutting insert, thus guaranteeing the alignment of the cutting and clamping parts of the tool. 

When observing the dynamics of the cutting forces, the oscillatory nature of the progress of the individual components was observed, which is influenced by several input factors. One influencing factor includes the possibility of improving the technological system, such as supporting the workpiece in the working space of the machine. This effect certainly influenced the overall quality of the machining process. The increase in the frequency of rotation of the machined material, which was limited by the mechanical equipment of the working area, would also have a positive effect. By increasing the workpiece rotation frequency, the tool rotation speed would be reduced, and the cutting conditions would be improved with respect to the cutting speed.

For AlZn5.5MgCu duralumin workpiece, the selected roughness parameters also increased with increasing speed. When the cutting speed was changed from 100 m·min^−1^ to 300 m·min^−1^, the increase in roughness parameters was low. At a cutting speed of 500 m·min^−1^, the roughness value increased exponentially by about 40%. The difference in surface texture at a cutting speed of 500 m·min^−1^ was also evident from the surface images (colour map of the surface—hexagonal texture). The measured parameters of the 42CrMo4 tough steel no longer increased upwards, but at a cutting speed of 300 m·min^−1^, the selected roughness parameters were lower than at 100 m·min^−1^. The highest measured roughness parameters were again found at a cutting speed of 500 m·min^−1^. Between the values of the cutting speed parameters of 300 m·min^−1^ and 500 m·min^−1^, there was again a step increase in the results. For hardened 90MnCrV8 steel hardened to 58 HRC, an upward increase in the evaluated roughness parameters with respect to cutting speed was observed. For this case, there was no stepwise increase in roughness. It can be concluded that the technology used is suitable for the application of machining quenched materials with respect to the roughness of the machined surface and the overall machining process. For quenched steel, better results of roughness parameters were obtained than for tough steel. The new monolithic rotary tool eliminates the high precision requirements for assembling complex tools. This can influence the resulting machined surface. Compared to self-propelled technology, this monolithic tool reduces the surface roughness of the machined surface *Rz* by 18 μm [25] and the value of *Ra* parameter is stabilised and less than 0.56–1.83 μm, which was achieved in other research [14]. With ADRT technology and the use of a multi-component tool, the roughness *Rz* 8 μm [7] was achieved, which is a higher value compared to the highest value measured after turning with the monolith rotary tool. Moreover, the roughness values *Ra* were achieved lower than with assembling complex tools. At identical feed rates, *Ra* varied from 1.14 μm to 2.09 μm, depending on the cutting parameters [23,26]. When applying the new monolithic tool, these *Ra* values were under 1 μm.

## 7. Conclusions

In the presented study, the suitability of the application of a new proposed monolithic tool designed for ADRT technology applications was investigated. Tools for such a kinematic scheme are currently not commercially available. Different prototypes of clamping systems for interchangeable cutting inserts and their actual production can be time-consuming and costly. Furthermore, setting the concentricity of the indexable cutting insert against the clamping part is demanding in terms of metrology equipment and personnel alone. This work aimed to verify a new proposed monolithic tool, which is patent pending. 

The given type of the proposed tool eliminates the shortcomings mentioned above of the design of tools assembled from multiple components. 

The identification of the applicability of the monolithic tool was carried out on three selected machined materials with different mechanical properties, representing the groups of tough and difficult to machine materials. 

For the basic determination of the cutting parameters, an experiment was carried out, which took into account the stiffness of the system and the possibilities of controlling the individual cutting parameters. Based on these data, the cutting parameters were determined and subsequently applied in the machining process. 

Experimental verification confirmed that the monolithic tool and the proposed technology are suitable for the implementation of high-speed machining under properly set cutting conditions for the reason that the maximum cutting force achieved did not reach such values that would result in fatal damage to the tool. 

Changes in the total cutting force as well as in the resulting surface roughness were observed when the tool rotation speed was changed. While the magnitude of the cutting force was most affected by the material type, where there is a noticeable increase in force from the deteriorating machinability of the material. For AlZn5.5MgCu and 90MnCrV8 materials, a decrease in cutting force can be observed as the tool rotation frequency increases. Higher tool rotation speed also had a positive effect on the resulting surface roughness, where for quenched steel 90MnCrV8, lower values of surface roughness parameters were obtained at a rotation speed of 500 m·min^−1^ compared to AlZn5.5MgCu and 42CrMo4 materials. 

The direction of tool rotation itself affects the overall cutting force in the machining process. Thus, with a properly selected rotation direction, a reduction in cutting force can be achieved, which in practice allows other cutting parameters to be adjusted in order to increase machining productivity.

Based on the presented results, it can be assumed that the new type of monolithic tool exhibits stable properties and that its use in broader engineering practice will be successful. The new monolith tool can be immediately applied to the production process while adhering to the kinematic scheme and adjusting the cutting parameters. It can also be used for hardened materials. 

In the future, it is possible to focus on machining nickel and titanium alloys, which are increasingly used outside the aviation industry. It is also important to focus on the tool life of the new monolith tool and the possibilities of cutting-edge renewal.

## Figures and Tables

**Table 1:** Chemical Composition 90MnCrV8 based on attestation certificate (wt%).

C	Si	Mn	P	S	Cr	V
0.85–0.95	0.1–0.4	1.9–2.1	0.03 max	0.03 max	0.2–0.5	0.05–0.15

**Table 2:** Chemical composition AlZn5.5MgCu based on attestation certificate (wt%).

Cr	Cu	Fe	Mg	Mn	Si	Ti	Zn
0.18–0.28	1.2–2	0.3 max	2.1–2.9	0.3 max	0.4 max	0.2 max	5.1–6.1

**Table 3:** Chemical composition 42CrMo4 based on attestation certificate (wt%).

C	Mn	Si	P	S	Cr	Mo	Cu
0.38–0.45	0.6–0.9	0.1–0.4	0.025 max	0.035 max	0.9–1.2	0.15–0.3	0.4 max

**Table 4:** Cutting parameters used for selected materials.

Material	Tool Cutting Speed *v_T_* [m·min^−1^]	Workpiece Rotation Speed *v_w_* [m·min^−1^]	Depth of Cut *a_p_* [mm]	Feed Rate *f* [mm]
42CrMo4	100	10	0.2	0.1
	300			
	500			
90MnCrV8	100			
	300			
	500			
AlZn5.5MgCu	100			
	300			
	500			

**Table 5:** Basic technical data for the three-component piezoelectric dynamometer.

Property	Numerical Value
Measuring range in the direction of *x*-axis	−20 to +20 kN
Measuring range in the direction of *y*-axis	−20 to +20 kN
Measuring range in the direction of *z*-axis	10 to 40 kN
Allowed operating temperature	0 to 70 °C
Actual frequency	3 kHz
Relative measurement uncertainty	1%
Measurement sensitivity	8 pC∙N^−1^

**Table 6:** Selected cutting parameters with respect to the system and machine stiffness.

*v_w_* (m·min^−1^)	*v_t_* (m·min^−1^)	*a_p_* (mm)	*f* (mm)
	50.00		
	100.00		
100.00	150.00	0.25	0.05
160.00	200.00	0.50	0.10
200.00	−50.00	0.75	0.15
	−100.00		
	−150.00		
	−200.00		

**Table 7:** Analysis of variance output for total cutting force F.

Source	DF	Contribution (%)	Adj SS	Adj MS	*F*-Value	*p*-Value
Model	11	90.6	823,072	74,825	32.53	0.000
Linear	5	34.21	310,365	77,591	39.96	0.000
*v_w_*	1	0.2	6320	6320	3.25	0.078
*v_t_*	1	8.83	75,248	75,248	38.75	0.000
*a_p_*	1	24.55	223,020	223,020	114.84	0.000
*f*	1	0.64	5776	5776	2.97	0.092
Square	4	55.78	506,762	126,691	65.24	0.000
v_w_ * v_w_	1	5.59	32	32	0.02	0.898
v_t_ * v_t_	1	34.76	315,782	315,782	162.61	0.000
a_p_ * a_p_	1	15.03	99,645	99,645	51.31	0.000
f * f	1	0.4	3640	3640	1.87	0.178
2-Way Interaction	3	0.61	5526	1842	0.95	0.425
v_w_ * v_t_	1	0	4	4	0	0.964
v_t_ * a_p_	1	0.18	1658	1658	0.85	0.361
v_t_ * f	1	0.43	3865	3865	1.99	0.165
Error	44	9.4	85,445	1942		
Total	55	100				

## Data Availability

The data that support the findings of this study are available from the corresponding author [RJ], upon reasonable request.

## References

[1] Kaulfersch F., Roeder M. (2013). Cutting of nickel-based superalloys with rotating indexable inserts. Adv. Mat. Res..

[2] Lei S., Liu W. (2002). High-speed machining of titanium alloys using the driven rotary tool. Int. J. Mach. Tools Manuf..

[3] Uhlmann E., Kaulfersch F., Roeder M. (2014). Turning of high-performance materials with rotating indexable inserts. Procedia CIRP.

[4] Yamamoto H., Satake K., Sasahara H., Narita T., Tsutsumi M., Muraki T. (2010). Thermal behavior and tool failures on rotary cutting of difficult-to-cut materials utilizing multi tasking lathe. Key Eng. Mater..

[5] Hosokawa A., Yoshimatsu H., Koyano T., Furumoto T., Hashimoto Y. (2018). Turning of difficult-to-machine materials with an actively driven rotary tool (ADRT)—Proposition of reciprocating turning contingent on fundamental cutting characteristics. J. Adv. Mech. Des. Syst. Manuf..

[6] Hosokawa A., Ueda T., Onishi R., Tanaka R., Furumoto T. (2010). Turning of difficult-to-machine materials with actively driven rotary tool. CIRP Ann. Manuf. Technol..

[7] Joch R., Pilc J., Stančeková D., Miturska I., Görögová I. (2020). Evaluation of Surface Roughness after Actively Rotary Turning Method. Mater. Sci. Forum..

[8] Ezugwu E.O. (2007). Improvements in the machining of aero-engine alloys using self-propelled rotary tooling technique. J. Mater. Process. Technol..

[9] Čep R., Janásek A., Martinický B., Sadílek M. (2011). Cutting tool life tests of ceramic inserts for car engine sleeves. Teh. Vjesn..

[10] Sasahara H., Kato A., Nakajima H., Yamamoto H., Muraki T., Tsutsumi M. (2008). High-speed rotary cutting of difficult-to-cut materials on multitasking lathe. Int. J. Mach. Tools Manuf..

[11] Ahmed W., Hegab H., Kishawy H.A., Mohany A. (2021). Estimation of temperature in machining with self-propelled rotary tools using finite element method. J. Manuf. Process..

[12] Nguyen T.T., Duong Q.D., Mia M. (2020). Sustainability-based optimization of the rotary turning of the hardened steel. Metals.

[13] Kishawy H.A., Ahmed W., Mohany A. (2021). Analytical modeling of metal cutting process with self-propelled rotary tools. CIRP J. Manuf. Sci. Technol..

[14] Ahmed W., Hegab H., Mohany A., Kishawy H. (2021). Analysis and Optimization of Machining Hardened Steel AISI 4140 with Self-Propelled Rotary Tools. Materials.

[15] Dessoly V., Melkote S.N., Lescalier C. (2004). Modeling and verification of cutting tool temperatures in rotary tool turning of hardened steel. Int. J. Mach. Tools Manuf..

[16] Zlámal T., Petrů J., Vortel O., Pagáč M., Krajkovič P. (2016). Mechanisms of cutting blade wear and their influence on cutting ability of the tool during machining special alloys. Adv. Sci. Technol. Res. J..

[17] Ekinović S., Dolinšek S., Begović E. (2005). Machinability of 90MnCrV8 steel during high-speed machining. J. Mater. Process. Technol..

[18] Isik Y. (2007). Investigating the machinability of tool steels in turning operations. Mater. Des..

[19] Lacková P., Žabecká D., Milkovič O., Škrobian M., Bajcura M. (2014). Effect of Technologies Processing on Material Properties of Selected Aluminium Alloys. Mater. Sci. Forum..

[20] Özdemir M., Kaya M., Akyildiz H. (2020). Analysis of surface roughness and cutting forces in hard turning of 42CrMo_4_ steel using Taguchi and RSM method. Mechanika.

[21] Bronis M., Miko E., Nowakowski L. (2021). Analyzing the Effects of the Kinematic System on the Quality of Holes Drilled in 42CrMo_4_ + QT Steel. Materials.

[22] Bajor T., Kulakowska A., Dyja H. (2020). Analysis of the rolling process of alloy 6005 in a three-high skew rolling mill. Materials.

[23] Olgun U., Budak E. (2013). Machining of difficult-to-cut-alloys using rotary turning tools. Procedia CIRP.

[24] Šajgalík M., Kušnerová M., Harničárová M., Valíček J., Czán A., Czánová T., Drbúl M., Boržan M., Kmec J. (2020). Analysis and prediction of the machining force depending on the parameters of trochoidal milling of hardened steel. Appl. Sci..

[25] Joch R., Pilc J., Daniš I., Drbúl M., Krajčoviech S. (2019). Analysis of surface roughness in turning process using rotating tool with chip breaker for specific shapes of automotive transmission shafts. Transp. Res. Proc..

[26] Akhyar G., Harun S., Hamni A. (2016). Surface Roughness Values of Magnesium Alloy AZ31 When Turning by Using Rotary Cutting Tool. Insist.

